# Deaf and hard-of-hearing children and adolescents’ mental health, Quality of Life and communication

**DOI:** 10.1186/s12888-023-04787-9

**Published:** 2023-04-28

**Authors:** Chris Margaret Aanondsen, Thomas Jozefiak, Stian Lydersen, Kerstin Heiling, Tormod Rimehaug

**Affiliations:** 1 Regional Centre for Child and Youth Mental Health and Child Welfare (RKBU Central Norway), Department of Mental Health, Faculty of Medicine and Health Sciences, NTNU – Norwegian University of Science and Technology, RKBU Midt-Norge, NTNU Postboks 8905 MTFS, 7491 Trondheim, Norway; 2 Unit for Deaf and Hard-of-Hearing Children and Adolescents in Central Norway, Department of Child and Adolescent Psychiatry, St. Olavs Hospital, Trondheim, Norway; 3 Hjärup, Sweden; 4 Department of Child and Adolescent Psychiatry, Nord-Trøndelag Hospital Trust, Levanger, Norway

**Keywords:** Mental health, Quality of Life, Deaf and hard-of-hearing children, Communication, Sign language

## Abstract

Mental health problems and lower Quality of Life (QoL) are more common in deaf and hard-of-hearing – (D)HH – children than in typically hearing (TH) children. Communication has been repeatedly linked to both mental health and QoL. The aims of this study were to compare mental health and QoL between signing deaf and hard-of-hearing (DHH), hard-of-hearing (HH) and TH children and to study associations between mental health/QoL and severity of hearing loss and communication. 106 children and adolescents (mean age 11;8; SD = 3.42), 59 of them DHH and 47 HH, and their parents reported child mental health and QoL outcomes. Parents also provided information about their children's communication, hearing loss and education while their children's cognitive ability was assessed. Although (D)HH and their parents rated their mental health similar to their TH peers, about twice as many (D)HH children rated themselves in the clinical range. However, (D)HH children rated their QoL as similar to their TH peers, while their parents rated it significantly lower. Associations between communicative competence, parent-reported mental health and QoL were found, whereas severity of hearing loss based on parent-report had no significant association with either mental health or QoL. These results are in line with other studies and emphasise the need to follow up on (D)HH children's mental health, QoL and communication.

## Background

For decades, mental health problems in deaf and hard-of-hearing "(D)HH" children and adolescents have been of clinical and research interest. In this paper, mental health problems are defined as the presence of symptoms of mental health disorders (e.g. low mood, problems with attention, etc.) as well as mental health disorders based on diagnostic classification, i.e. the combination and severity of symptoms combined with clinically significant loss of function. In the following sections, the term "HH" refers to hard-of-hearing children with a preference for spoken language; "DHH" to signing deaf and hard-of-hearing children and "(D)HH" to both groups, while the term "children" will describe both children and adolescents in this paper.

### Mental health

Elevated prevalence of mental health problems has been reported for (D)HH children; twice to four times as high as their typically hearing (TH) peers depending on other risk factors [1–10]. These findings are mainly based on parent and teacher-report, whereas (D)HH children do not rate themselves significantly higher than their TH peers on mental health problems [2, 3, 11]. A systematic review on psychopathology [11] reports more symptoms of depression, anxiety, aggression and behavioural disorders in (D)HH children than in TH children. Good communication skills, early detection of hearing loss (HL), early intervention, good cognitive abilities, good peer relationships for girls and good sports skills for boys have been identified as potential protective factors for mental health problems [1, 5, 10, 11]. Aetiology of HL, additional disabilities, intellectual impairment and low language abilities have been established as risk factors for mental health problems [5, 8, 12]. As many as 25% to 40% of (D)HH children have additional disabilities [4, 5, 13]. Degree of HL has not been found to have an effect on mental health problems [11]. Late or incorrect diagnosis of mental health problems in (D)HH people is common due to the complexity and overlap of cultural, linguistic and clinical factors [14–17]. A recent study on psychiatric disorders and reasons for referral to generic Child and Adolescent Mental Health Services (CAMHS) showed that 18.1% of the (D)HH children in Norway were referred compared to 5% of the TH population [18]. Norwegian DHH children were also referred earlier than their TH peers.

### Quality of life

Comparing studies on Quality of life (QoL) is challenging due to the variety of definitions of the concept. The World Health Organisation (WHO) defines QoL as “an individual's perception of their position in life in the context of the culture and value systems in which they live and in relation to their goals, expectations, standards and concerns” (World Health Organization, 1995, p. 1405 [19]). Quality of Life (QoL) in (D)HH children, especially those using cochlear implants (CI), has been a point of interest for researchers from several fields. A systematic review on QoL after paediatric CI implantation [20] concludes that few of these studies are based on generic measures and primarily on small sample sizes, precluding other overall findings on QoL. The same authors, therefore, recommend strict inclusion criteria and generic QoL instruments for future studies. Another systematic review [21] finds that (D)HH children score significantly lower on validated QoL measures. Methodological issues with this systematic review, including misinterpretation of the results of two of the included studies, have been discussed by Aanondsen, Jozefiak, Heiling et al. [22]. Some studies report no significant differences between TH and (D)HH children in QoL [23–25], whereas recent Norwegian studies on (D)HH children [3] and children with CI [26, 27] report significantly lower overall QoL (parent and self-report). The subjectiveness of QoL as a concept [28], differences between parent- and self-report, and the importance of multiple perspectives have been described [21, 22, 29–34]. Age [31, 35], degree of HL [23–25], and communication [26, 27, 36] have all been associated with QoL in (D)HH children. Significant negative associations between age and QoL for TH [37] and (D)HH children [31, 35] have been confirmed, whereas negative associations between degree of HL and QoL have not [23–25].

### Communication

In this paper, the term “communication” will refer to language skills and communicative competence, whereas “language” refers to all natural languages, i.e., both spoken and sign languages independent of modality and country unless otherwise specified; when referring to other studies the respective authors’ terms are used for vocabulary, pragmatic skills, social communication, etc.

Mode and level of communication have been suggested as factors affecting mental health and QoL in (D)HH children. The historical debate about sign versus spoken language for (D)HH children has been reignited by technological advances such as CIs [38], and bilingual/bicultural approaches have been questioned again [39]. Even though CIs have improved speech and spoken language outcomes, (D)HH children experience delays in language development [26, 40–42]. Sign languages have been acknowledged as natural languages [43], leading to a shift viewing (D)HH people in a socio-cultural instead of a disability perspective [44]. There is currently no consensus on interventions [38] due to variability in language outcomes [45], small sample sizes [38], the lack of studies on functional language, and focus on vocabulary, speech perception and production [39, 46]. As only about 5% have (D)HH parents [47], most (D)HH children learn sign language from non-native signers, which can cause delays [48]. DHH children of DHH parents reach language milestones as quickly as TH peers [47–49]. Successful communication between parents and (D)HH children is essential for language, cognitive and socio-emotional development independent of modality [40, 50].

Good communication skills independent of modality are associated with better mental health [1, 10, 11], while negative associations have been found for language development and behavioural problems [12, 51]. In addition, negative associations have been found for (D)HH children’s functional language skills in conversation with TH peers and peer problems [52]. Ching, Cupples, Leigh et al. [53] report positive associations between functional auditory performance, use of speech and language skills, psychosocial skills, and QoL and therefore emphasise the importance of functional performance. The same authors also found that better auditory functional performance and pragmatic language skills were associated with better psychosocial abilities and QoL, whereas structural language abilities were not [53].

Overall, several factors complicate the understanding of mental health, QoL and communication in (D)HH children and adolescents. Among these are the low prevalence of moderate to profound HL [54], resulting in small sample sizes, heterogeneity in outcomes for mental health, QoL [20] and communication due to differences in aetiology of HL [55], access to newborn hearing screening, early intervention as well as the likelihood of other disabilities [4, 9]. In addition, mode and level of communication [1, 11], differences in services between countries, choice of instruments (generic vs ad hoc [20], written vs sign language measures [14, 56]) as well as methodological issues such as those reported here previously [22] also contribute to the heterogeneity in findings. Assessing DHH children is challenging, as few validated assessment tools are available in sign language, but necessary as studies have shown that DHH children report more symptoms on measures in Sign Language than on written ones [14, 56].

This study addresses several of the challenges outlined above by assessing mental health problems and QoL in DHH children with validated questionnaires in both written Norwegian and Norwegian Sign Language (NSL). It also provides insight into the mental health and QoL of Norwegian (D)HH children and adolescents. Knowledge of the prevalence of mental health problems can in turn be used to improve early intervention and the organisation of specialised mental health services in Norway.

## Aims

The main aim of the present study is to study associations between mental health, QoL, communication, and parent-reported HL in hard-of-hearing (HH) and signing DHH children.

We addressed the following research questions:

Are there differences in mental health (self-and parent-report) between (D)HH and TH children?
Are there differences in QoL (self-and parent-report) between (D)HH and TH children?
A) What is the association between (D)HH children's degree of HL and mental health?
  B) What is the association between (D)HH children's degree of HL and QoL?
A) What is the association between (D)HH children's communication (language skills and communicative competence) and mental health?
  B) What is the association between (D)HH children's communication (language skills and communicative competence) and QoL?

## Methods

### Participants

**Fig. 1:**
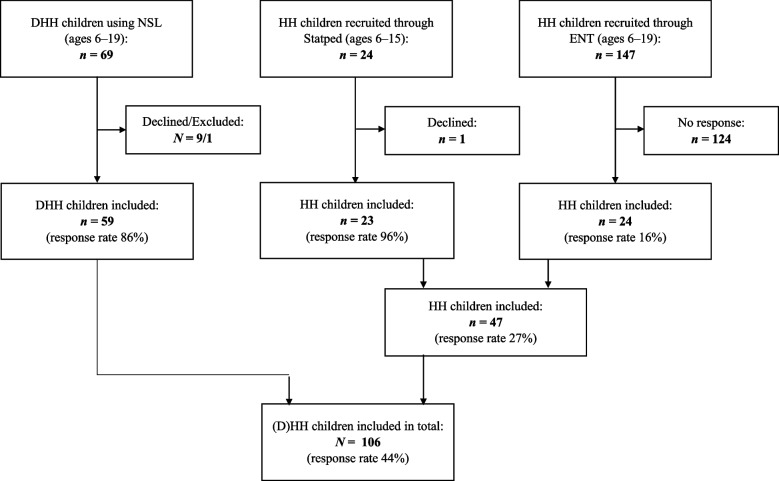
Flow chart for the inclusion of participants

Hearing loss of > 40 dB affects 1.4 per 1000 infants [57]; this amounts to 266 children aged 6–18 with a HL in central and northern Norway. DHH children aged 6–17 were recruited from A.C. Møller school, a deaf school for central and northern Norway. DHH adolescents aged 15–20 attending Tiller Upper Secondary School (Trondheim, Norway) with NSL as their first or second language were also invited. In total, 86% (59/69) of the DHH children were included. Ninety-six percent (23/24) of the HH children aged 6–15 were recruited from the Norwegian National Support System for Special Education (Statped), and 16% (24/147) of the HH children aged 6–19 from the local audiology department. None of the HH children received their education in Norwegian Sign Language either part- or full time. The overall response rate for the complete sample (DHH and HH children) was 44% (106/240) (see Fig. 1). The parents of all children were also invited to participate.

In total, 106 (D)HH children (62.3% girls) participated, with a mean age of 11.8 years (SD = 3.42; range 6 to 19) and a mean nonverbal IQ of 108.0 (SD = 15.46; range = 74–143). One participant with a very low non-verbal IQ was excluded from analysis. Among the DHH children, 45 DHH children completed the SDQ-NOR, whereas 40 completed both the SDQ-NSL and the SDQ-NOR. For the ILC, 55 DHH children completed the ILC-NOR and 48 completed both the ILC-NSL and the ILC-NOR. Seventy-three of the 86 (84.9%) mothers had completed at least 12 years of education, whereas 56 of the 82 (68.3%) fathers had completed at least 12 years of education. Data were collected between November 1, 2016, and March 23, 2018. The majority of the DHH children (73.2%) mainly attended mainstream schools while spending two to six weeks at the deaf school per school year. Almost all HH children (87%) attended mainstream school full-time. The participants' hearing and communication-related information are reported in Tables 1 and 2.

**Table 1:** Hearing-related characteristics (parent report) for Norwegian DHH and HH children

Variable	DHH *n* = 41	%	HH *n* = 45	%
(D)HH parent				
Yes / No	7/34	20.6/79.4	5/40	11.1/88.9
Time in deaf school ^a^				
2–6 weeks a year	30	73.2	6	13.3
> 7 weeks a year	7	20.6	0	0
1–2 days a week ^b^	7	20.6	2	4.4
5 days a week	4	9.5	0	0
Aetiology of hearing loss				
Acquired	4	9.8	8	17.8
Congenital	36	87.8	33	73.3
Unknown	1	2.4	4	8.9
Missing	0	0	0	0
Degree of hearing loss				
Moderate: 40–70 dB	10	24.4	23	51.1
Severe: 71–100 dB	14	34.1	7	15.6
Profound: 101 +	12	29.3	3	6.7
Unknown	5	12.2	9	20
Missing	0	0	3	6.7
Use of hearing aid (Yes / No) ^c^				
CI	19/22	44.3/53.7	3/42	6.7/93.3
Hearing aid	33/ 8	80.5/19.5	38/7	84.4/15.6
Missing	0	0	0	0
Age at detection				
0–2 years	26	63.4	19	42.2
3–5 years	15	36.6	19	42.2
6–12 years	0	0	6	13.3
Unknown	0	0	1	2.2
Preferred language				
Oral	21	51.2	40	88.9
Sign	5	12.2	1	2.2
Bilingual	15	39.0	3	6.7
Missing	0	0	1	2.2
Other impairment ^d^				
Vision	14	34.1	11	24.4
Motor	1	2.4	2	4.4
Learning	4	9.8	4	8.9
Other	8	19.6	4	8.9
Missing	2	4.9	1	2.2

41 of the 59 parents of DHH children completed the parent-report; 9 of the missing parent-reports were due to adolescents aged 16 or older not consenting to their parents’ participation; 45 of the 47 parents of the HH children completed the parent-report on hearing-related characteristics

^a^ All children attended both mainstream and deaf school

^b^ Children attending the deaf school for 1–2 days a week combine this with two or more week-long stays during the school year; that is, total number of answers is greater than the number of participants

^c^ Based on reports of ever having used a hearing aid

^d^ Some of the children had more than one impairment

**Table 2:** Communicative competence (parent report)

	DHH		HH	
Communication Skills	*n*	M (SD)	*n*	M (SD)
Sign Language Skills (1–12)	38	9.11 (2.14)	4	8.50 (4.04)
Spoken Language Skills (1–12)	40	11.20 (1.70)	36	11.61 (1.13)
CCC-2 GCC (0–168)	35	53.26 (21.95)	38	62.32 (24.01)

Sign Language Skills based on the sum scores of the sign language production scale (SPS) and the sign language understanding scale (SUS); range 1–12. Higher scores indicate better sign language skills

Spoken Languages Skills based on the sum scores of Categories of Auditory Performance (CAP) and Speech Intelligibility Rating (SIR); range 1–12. Higher scores indicate better spoken language skills

CCC-2: Children's Communication Checklist version 2; GCC: General Communication Composite; range 0–168; a GCC score of 83 equals the mean or 51st percentile, while a GCC score of 61 equals the 15th percentile [64]

Studies based on Norwegian community samples, employing the same measures as in our study, were used for comparison. Data for the study on the SDQ-NOR were collected in 2002 [58, 59]. The self-report sample included 29426 [58] TH children aged 8 to 19, while the parent-report sample included 8517 parents of TH children [59] aged 10 to 13. Data for the ILC-NOR were collected between 2004 and 2005, for TH children aged 8 to 16, which resulted in 1987 self-reports and 2563 parent-reports [30].

### Measures

#### Socio-demographic and hearing-related information

Parents completed a questionnaire about their children’s age and gender, type of education, and their own attendance of sign language classes. Parents also reported on their children’s type and degree of HL, as we did not have access to audiological records,

### Communication

#### Spoken language skills

Auditory performance (speech intelligibility and listening skills) were assessed using Categories of Auditory Performance (CAP; [60]) and Speech Intelligibility Rating (SIR, [61]). The CAP and SIR are validated single-item scales frequently used in research. Interrater reliability for the Danish version was reported as good (CAP: kappa = 0.785; SIR: kappa = 0.848 [1]). The sum of the CAP and SIR was calculated for each child as the Spoken Language Skills Score.

#### Sign language skills

Sign language skills were assessed using the Norwegian version of the Sign Language Production Scale (SPS) and the Sign Language Understanding Scale (SUS). Dammeyer [1] designed and validated the SPS and SUS as a short screening of sign language skills for research purposes based on the CAP and SIR. The interrater reliability of the Danish version was reported as being good (kappa = 0.944 for SUS and kappa = 0.921 for SPS; [1]). The validity of the Danish version of the SUS based on correlations between the SUS and the sign language receptive skill test reached statistical significance (Spearman rank correlation coefficient = 0.905, *p* < 0.000;). No corresponding test was available for sign language production. The sum of the SPS and SUS scores, the “Sign Language Skills Score,” was calculated for each child.

#### Communicative competence

The participants' communicative competence was assessed using the Norwegian version [62] of the Children’s Communication Checklist Second Edition (CCC-2), which was developed by Bishop [63, 64]. The reliability and validity of the Norwegian version have been confirmed [65]. The CCC-2 is completed by parents and/or teachers. It consists of 10 subscales (7 items per subscale): (A) speech, (B) syntax, (C) semantics, (D) coherence, (E) inappropriate initiation, (F) stereotyped language, (G) use of context, (H) non-verbal communication, (I) social relations, and (J) interests. The General Communication Composite (GCC) is based on the sum of the scaled scores for subscales A to H.

### Cognitive abilities

The nonverbal intelligence of the participants was assessed using the well-validated Leiter International Performance Scale – Third Edition (Leiter-3) [66]. Compared to the original validation, significantly higher nonverbal IQ scores were found for a TH Scandinavian sample (M = 108.6, SD = 8.4; [61]).

### Mental health

The Strengths and Difficulties Questionnaire (SDQ) is a brief well-validated [67, 68] measure to assess mental health problems and pro-social behaviour. It consists of 25 items, each scored on a 3-point Likert scale (0 = "Not true," 1 = "Somewhat true" and 2 = "Certainly true"). These are grouped into five scales (Emotional Problems, Conduct Problems, Hyperactivity-Inattention, Peer Problems, and Pro-social Behavior) as well as a Total Difficulties score based on the four negative subscales, higher scores indicating more difficulties. For this study, we administered both the parent and the self-report versions of the SDQ in written Norwegian (SDQ-NOR) and in Norwegian Sign Language (SDQ-NSL; [69]); self-reports only for children ≥ 9.

### Quality of Life (QoL)

The Inventory of Life Quality in Children and Adolescents – ILC [30, 32] is a validated multi-informant assessment for QoL based on seven items. One item assesses overall QoL, and six items address the child's physical and mental health, school and family functioning, social contact with peers, interests, and recreational activities. [30, 32]. We administered both the parent and the self-report versions of the ILC in written Norwegian (ILC-NOR) and NSL (ILC-NSL; [22]) for this study.

### Procedures

The DHH children and their parents received oral/signed and written information about participation. According to the study's survey procedures, written informed consent was obtained for all participants prior to inclusion. I.e. from all parents as well as from adolescents ≥ 16. DHH children ≤ 16, whose parents already had consented to their children’s participation, were also asked to confirm consent verbally or in NSL. DHH children responded to the written and the NSL versions of the ILC and SDQ. A more detailed description of the DHH group can be found here: [22, 69]. The HH children and their parents recruited through the ENT department received information about the study by mail. According to the study's survey procedures, written informed consent was obtained from adolescents ≥ 16 and parents before inclusion. The HH children responded to the paper and pencil version of the SDQ-NOR and ILC-NOR. Parents for both groups completed questionnaires on language, communicative competence, HL, education, and the SDQ and ILC.

### Statistical analyses

We handled missing values using multiple imputation; a detailed description of the missing values can be found in Appendix 3. The imputation model included all variables used in at least one of the subsequent analyses. Degree of HL, additional impairment, total score SDQ (parent and self-report: SDQ-NSL and SDQ-NOR), QoL score (parent and self-report: ILC-NSL and ILC-NOR), CCC-2 GCC, and spoken and sign language skills were included. The following variables were also included in the imputation model: group (DHH/HH), gender, age, mother's and father's education, nonverbal IQ, age at detection of HL and cause of HL. We created M = 100 imputed data sets, generally regarded as sufficient [70]. We imputed with no restrictions to the range and no post-imputation rounding, as recommended [71]. Analysis based on multiple imputation provides unbiased estimates under the missing at random assumption, while a complete case analysis would give unbiased estimates only under the more restrictive missing completely at random assumption. Some variables were structurally missing, such as CCC-2 GCC for those who do not speak in complete sentences, SDQ-NSL, ILC-NSL and sign language skills for those not using NSL. First, we imputed all missing values, including these, then we deleted the imputed values in the positions where there were structurally missing.

Associations between HL, communication, mental health and QoL were investigated using linear regression with mental health and QoL as dependent variables. All regression analyses were adjusted for age and gender. Group differences between DHH and TH children were studied using t-tests not assuming equal variances. Wald confidence intervals were calculated for proportions. Two-sided *p* values < 0.05 were taken to indicate statistical significance, and 95% confidence intervals (95% CI) are reported where relevant. However, *p*-values between 0.01 and 0.05 should be interpreted with caution due to multiple hypotheses. All analyses were conducted in Stata/SE 17.0 for Windows.

### Ethics

Before inclusion, written informed consent was obtained from the parents and adolescents ≥ 16 and oral/signed informed consent from the children < 16. Study approval was given by the Regional Committees for Medical and Health Research Ethics (reference number: 2015/1739/REK midt).

## Results

### Mental health in DHH, HH and TH children

No significant differences were found between DHH and HH children for self-reported (ß.-0.672; CI: -4.36 to 3.01; p:0.715) and parent-reported mental health (ß -0.569; CI: -3.20 to 2.06; p: 0.668). DHH children reported significantly more mental health symptoms on the SDQ-NSL than TH children on the SDQ-NOR. For the SDQ-NOR, no significant differences were found between DHH and TH children and HH and TH children on both self- and parent-report (Table 3).

**Table 3:** Comparison of DHH, HH and TH children's mental health (means and SD) based on multiply imputed data

		DHH^2^		HH^2^		TH (van Roy et al. [58, 59])^1^	DHH – TH^5^		HH – TH^6^	
	*n*	*M (SD)*	*n*	*M (SD)*	*n*	*M (SD)*	95% CI	*p*	95% CI	*p*
SDQ-NOR	*48*	12.05(6.40)	*37*	10.98 (6.81)	29,426	10.65 (5.5)^3^	-.46 to 3.26	.137	-1.94 to 2.60	.770
SDQ-NSL	*48*	13.24 (6.61)		N/A		N/A	**.67 to 4.51**	**.009**	N/A	N/A
SDQ P	*59*	7.25 (5.97)	*47*	6.48(5.60)	8517	6.15 (5.0)^4^	-.46 to 2.66	.163	-1.32 to 1.98	.689

SDQ Strengths and Difficulties Questionnaire; range of total score 0–40; SDQ-NSL: SDQ self-report in Norwegian Sign Language. SDQ-NOR: SDQ self-report in written Norwegian. N/A: not applicable

^1^ TH SDQ total scores from other authors community studies

^2^ No significant differences between DHH and HH based on linear regression analysis with mental health as dependent variable and group (DHH/HH) as independent variable. Regression analyses are adjusted for age and gender and based on multiply imputed data

^3^ Community sample self-report [58]; *n* = 29,426; age range 8 to 16

^4^ Community sample parent-report [59]; *n* = 8517; age range 10 to 13

^5^ T-tests not assuming equal variances for differences between DHH and TH; TH data on the SDQ-NOR were used for comparison with the DHH children on both the SDQ-NOR and SDQ-NSL

^6^ T-tests not assuming equal variances for differences between HH and TH

Eighteen point two percent of the DHH children rated themselves in the clinical range on the SDQ-NSL, 16.4% on the SDQ-NOR and 16.2% of the HH children compared to 8.7% of the TH children [58]; see Table 4. Prevalence based on complete case analysis can be found in Appendix 1.

**Table 4:** Prevalence of mental health problems in DHH, HH and TH children based on multiply imputed data (MI). Estimates and 95% confidence intervals (CI) based on Wald

	DHH				HH		TH (van Roy et al. [58])
Classification %	SDQ-NOR		SDQ-NSL		SDQ-NOR		SDQ-NOR
Type of data	MI	CI	MI	CI	MI	CI	
Normal	76.5	63.6 to 89.3	66.1	51.0 to 81.2	68.7	50.7 to 86.5	82.2
Borderline	7.1	0.7 to 15.0	15.7	4.0 to 27.3	15.1	1.7 to 28.6	9.1
Clinical	16.4	5.2 to 27.7	18.2	5.6 to 30.9	16.2	2.0 to 30.5	8.7

SDQ Strengths and Difficulties Questionnaire; total score range 0–40; SDQ-NSL: SDQ self-report in Norwegian Sign Language (NSL). SDQ-NOR: SDQ self-report in written Norwegian; prevalence based on Norwegian cut-off scores for the self-report [83]

MI: multiply imputed data

### QoL in DHH, HH and TH children

No significant differences were found between DHH and HH children for self-reported (ß 0.393; CI: -1.33 to 2.12; p:0.651) and parent-reported QoL (ß 1.03; CI: –0.92 to 2.98; p: 0.297). Parents of DHH and HH children reported significantly lower QoL than parents of TH children: see Table 5.

**Table 5:** Comparison of DHH, HH and TH children's QoL (means and SD) based on multiply imputed data

		DHH		HH		TH (Jozefiak et al. [30])^1^	DHH – TH^3^		HH – TH^4^	
	*n*	*M (SD)*	*n*	*M (SD)*	*n*	*M (SD)*	95% CI	*p*	95% CI	*p*
ILC-NOR	*59*	21.89 (4.64)^2^	*47*	22.84 (3.97)	*1987*	22.23 (3.93)	-1.56 to .88	.580	-.57 to 1.79	.303
ILC-NSL	*59*	21.04 (4.80)^2^		N/A	*1987*	N/A	-2.45 to .07	.064	N/A	N/A
ILC-P	*59*	21.45 (4.65)^2^	*47*	22.96 (4.25)	*2563*	24.71 (2.98)	**-4.48 to -2.04**	** < .001**	**-3.00 to -.50**	**.007**

ILC: The Inventory of Life Quality in Children and Adolescents (ILC): QoL score (LQ0-28): range 0–28, 28 = high QoL

^1^TH ILC QoL score from other authors community studies; Community sample self- (*n* = 1987) and parent-report (*n* = 2563), age range: 8 to 16; [30]

^2^No significant group differences based on linear regression analysis with QoL as dependent variable and group (DHH/HH) as independent variable. Regression analyses are adjusted for age and gender; analyses are based on multiple imputation

^3^ T-tests not assuming equal variances for differences between DHH and TH; TH data on the ILC-NOR were used for comparison with DHH children on the ILC-NOR and ILC-NSL

^4^ T-tests not assuming equal variances for differences between HH and TH

N/A – not applicable

### Associations between mental health, QoL and HL in DHH and HH children

Small and non-significant regression coefficients were found for degree of HL on mental health (see Table 6) and QoL (see Table 7) for both self- and parent-reports.

**Table 6:** Linear regression with mental health as dependent variable, HL, language skills or communicative competence added one at a time as covariates based on multiply imputed data

		Degree of HL				Spoken Language Skills				Sign Language Skills				CCC-2		
	*n*	*ß*	95% CI	*p*	*n*	*ß*	95% CI	*p*	*n*	*ß*	95% CI	*p*	*n*	*ß*	95% CI	*p*
SDQ-NOR	*85*	-.02	-.09 to .06	.634	*85*	-.27	-1.86 to 1.33	.734	*48*	-.46	-1.55 to .63	.392	*77*	-.04	-.13 to .04	.317
SDQ-NSL	*48*	.01	-.11 to .13	.880	*48*	-.27	-2.16 to 1.62	.766	*48*	-.69	-1.82 to .43	.212	*43*	-.08	-.20 to .04	.189
SDQ P	*106*	-.04	-.09 to .01	.089	*106*	-.54	-1.56 to .48	.292	*59*	-.22	-1.16 to .72	.636	*97*	**-.10**	**-.15 to -.05**	** < .001**

SDQ Strengths and Difficulties Questionnaire; total score range 0–40; SDQ-NSL: SDQ self-report in Norwegian Sign Language (NSL). SDQ-NOR: SDQ self-report in written Norwegian; SDQ-P: parent report

CCC-2: General Communication Composite of the Children's Communication Checklist Second Edition (GCC)

All analyses are adjusted for age and gender. *ß* regression coefficient

**Table 7:** Linear regression with QoL as dependent variables, HL, language skills or communicative competence added one at a time as covariates based on multiply imputed data

		Degree of HL				Spoken Language Skills				Sign Language Skills				CCC-2		
	*n*	*ß*	95% CI	*p*	*n*	*ß*	95% CI	*p*	*n*	*ß*	95% CI	*p*	*n*	*ß*	95% CI	*p*
ILC-NOR	*106*	.20	-.02 to .06	.344	*106*	.08	-.59 to .75	.806	*59*	***.60***	***.01 to 1.18***	***.05***	*97*	.02	-.03 to .07	.396
ILC-NSL	*59*	.02	-.05 to .09	.596	*59*	.43	-.59 to 1.46	.392	*59*	.51	-.37 to 1.38	.238	*53*	.05	-.02 to .13	.141
ILC-P	*106*	.02	-.02 to .05	.420	*106*	.53	-.17 to 1.24	.136	*59*	.31	-.47 to 1.09	.414	*97*	**.10**	**.06 to .14**	** < .001**

ILC: The Inventory of Life Quality in Children and Adolescents; QoL score LQ_0-28_: range 0–28, 28 = high QoL; ILC-NSL: ILC self-report in NSL

ILC-NOR: ILC self-report in written Norwegian; ILC-P: parent-report

CCC-2: General Communication Composite of the Children's Communication Checklist Second Edition (GCC)

All analyses are adjusted for age and gender. *ß* regression coefficient

### Associations between mental health, QoL and communication in DHH and HH children

Significant regression coefficients were found for communicative competence (CCC-2) and parent-reported mental health (see Table 6), and parent-reported QoL (see Table 7). For spoken and sign language skills, however, non-significant regression coefficients were found for spoken and sign language skills on mental health (see Table 6) for both self-and parent-report. For QoL a significant regression coefficient was found for sign language on the ILC-NOR (see Table 7), but not for sign language on the ILC-NSL. 

## Discussion

In the present study, no mean differences were found between DHH, HH and TH children for self– or parent-reported mental health on the SDQ-NOR. On the SDQ-NSL, however, DHH children reported significantly more mental health problems than TH children, and more than twice as many DHH children scored in the borderline range on the SDQ-NSL compared to the SDQ-NOR. Both DHH and HH children were about twice as likely to rate themselves in the clinical range for mental health problems compared to TH children, indicating clinically significant differences in mental health. As the data for the TH children were collected 15 years prior to our study, this could cause concerns regarding the validity of our comparison as an increase of emotional problems, especially in teenage girls has been reported both nationally [72, 73] as well as globally [74]. There are, however, other studies such as the one by Pitchforth, Fahy, Ford et al. [75] on a large national British sample that did not find an increase in mental health problems based on the SDQ total score for children and adolescents aged 4 to 16 between 1995 and 2014. As the SDQ total score is based on both internalising and externalising problems and studies such as the one by Pitchforth, Fahy, Ford et al. [75] do not report a significant increase, differences in prevalence for mental health problems found in this study are not likely to be explained solely by an increase in emotional problems in general.

Parents of DHH and HH children reported significantly lower QoL than parents of TH children, while DHH and HH children did not rate themselves significantly differently from their TH peers. Degree of HL was not associated with either mental health or QoL, whereas parent-rated communicative competence was positively associated with parent-rated mental health and QoL.

The lack of differences in mental health between DHH and HH children [2, 7, 76] and the lack of association between degree of HL and mental health are in accordance with previous studies [1, 11, 77–79]. The elevated prevalence is also in accordance with several international studies [1, 2, 6, 9, 11] and two recent Norwegian [3, 18] studies, although some report a prevalence of four times as high.

DHH children being more likely to rate themselves in the borderline range for mental health problems on the SDQ-NSL is in accordance with other studies that have found DHH children to under-report symptoms on written measures [14, 56]. Parental support, including sign language tuition, is quite extensive in Scandinavia, which may result in better mental health in (D)HH children, as suggested by other authors [4]. This is likely to have had a preventive effect on mental health [5, 11, 80].

The (D)HH children in this study did not rate their QoL as significantly different from TH peers, which was in line with studies from other countries [23, 24, 29], but in contrast to two Norwegian studies [3, 26]. Overgaard, Oerbeck, Wagner et al.’s study [3] only included self-report measures for adolescents ≥ 13, the (D)HH children in our study were younger. As age has been found to be negatively associated with QoL, this could explain the difference. A closer look at the mean scores of Overgaard, Oerbeck, Wagner et al. [3]’s study shows that the self-reported mean scores of the DHH children were in the average range according to the Norwegian norms, indicating that the observed difference is statistically but not clinically significant. The parents in our study rated QoL of their DHH and HH children as significantly lower than parents of TH children. Parents of DHH children scored their children’s QoL as close to the “below average” range according to Norwegian norms, indicating a clinically significant difference [30]. The difference in ratings between parents and children as informants has been observed in several studies [22, 29, 34, 81]. Possible explanations for this might be that parents experience the impact of their child's HL as more severe [31], that some aspects of QoL (school, family, friends) are less observable for parents [34] or that communication problems may prevent parents from a better insight into their children's subjective experience [29]. The difference in perspectives, which also is reported in TH children, emphasises the need to use self-reported QoL as the authentic QoL report, to evaluate (D)HH children's QoL, QoL being a subjective concept per definition. Parent-report should be used as supplemental information only [28].

Significant associations between communicative competence, mental health and QoL were found for parent-reported mental health and QoL. A closer look at other studies that have found associations between (D)HH children's communication skills and mental health showed that these studies were based on either parent- and/or teacher-report, but not self-report [1, 6, 8]. Our findings are, therefore, in line with these. Spoken and sign language skills not showing significant associations with parent-reported mental health was more surprising as we used the same assessment tools as Dammeyer [1] and a similar sample. A possible explanation could be the statistics used, where Dammeyer [1] dichotomised SDQ-total scores and looked at the (D)HH children with high language skills (maximum score only); or generally high performance, especially on spoken language skills for (D)HH children in his study. The significant positive associations between parent-reported sign language skills and self-reported QoL are in accordance with two studies [26, 27] that find weak to moderate significant correlations between language skills and QoL. When adolescents perceive to understand most of their parents' expressive communication, they report better QoL [36]. Several authors have noted the lack of studies based on pragmatic/functional language [39, 46] use, while others have shown that (D)HH children with age-appropriate vocabulary still are delayed in pragmatic language development [82]. This may explain the variations in findings [10, 80] and emphasises the need for validated tools for pragmatic language and communicative abilities for (D)HH children.

### Strengths and limitations

A major strength of the present study is the use of generic assessment tools (SDQ and ILC) also validated in NSL. Further strengths are the multi-informant perspective, a representative sample for DHH children for our region and information on HL, additional disabilities, cognitive abilities, communication, age at detection and type of school. A major limitation is the small sample size that prevented us from studying relevant subdomains within mental health, QoL and communicative competence, and affects statistical precision and generalisability. Another limitation is the low participation of HH children recruited from the ENT unit, which affects the representativeness of the HH group. A further limitation is related to the comparison of the TH samples, as the studies on the SDQ and the ILC for these samples were carried out 10 to 15 years prior to our study as already discussed regarding the SDQ. For the comparison of the ILC this does not pose a problem as a recent Norwegian study confirmed that QoL-reports in adolescents have been stable across the past 13 years [73]. Further, the spoken and sign language measures are simple one-scale items that do not capture the complexity of pragmatic language. The CCC-2 is not designed or validated for (D)HH children but was used as the best solution at the time of data collection to collect data on pragmatic language and compensate for single-item scales of the CAP and SIR. As parents were the only raters to report on both communication and mental health/QoL, this might have affected the associations found in this study. An objective assessment of communication could have compensated for this.

## Conclusion

Although (D)HH children and their parents, on average, rate their mental health as similar to their TH peers, about twice as many rate themselves in the clinical range for mental health problems. Even though prevalence is lower than in international studies, it still emphasises the need for specialised mental health services and validated assessment tailored to this group to ensure early referral and correct assessment. (D)HH children do not rate their QoL as lower than their TH peers, in contrast to their parents. This emphasises the importance of using parent-reported QoL as supplementary information only. The associations between communicative competence, mental health and QoL capture the complexity of understanding and supporting (D)HH families, the need to consider overall development and interdisciplinary collaboration. The need for validated assessment of pragmatic language skills (spoken and sign) for Norwegian (D)HH children has also become apparent for clinical and research purposes. International longitudinal interdisciplinary studies and systematic reviews should be conducted to handle challenges with small sample sizes, cross-sectional studies, and lacking consensus.

## Data Availability

The datasets generated and/or analysed during the current study are not publicly available due to the sensitivity of information and the small sample size. They are available from the corresponding author upon reasonable request.

## Appendix 1

**Table 8:** Table A Prevalence of mental health problems in DHH, HH and TH children based on complete case analysis (CC) data. Estimates and 95% confidence intervals (CI) based on Wald

	DHH				HH		TH (van Roy et al. (58))
Classification %	SDQ-NOR		SDQ-NSL		SDQ-NOR		SDQ-NOR
Type of data	CC	CI	CC	CI	CC	CI	
Normal	77.8	65.3 to 90.3	69.2	54.3 to 84.2	66.6	46.8 to 86.6	82.2
Borderline	6.7	0.8 to 14.2	15.4	3.7 to 27.1	16.7	0.9 to 32.4	9.1
Clinical	15.5	4.7 to 26.4	15.4	3.7 to 27.1	16.7	0.9 to 32.4	8.7

SDQ Strengths and Difficulties Questionnaire; total score range 0–40; SDQ-NSL: SDQ self-report in Norwegian Sign Language (NSL). SDQ-NOR: SDQ self-report in written Norwegian; prevalence based on Norwegian cut-off scores for the self-report (83)

CC: complete case analysis

## Appendix 2

**Table 9:** Norwegian version of the Sign Language Understanding Scale (SUS) VTF—Vurdering av tegnspråklig forståelseSett et kryss ved nivået, som passer best på barnet

0	Registrerer ikke eller oppfatter ikke tegn
1	Registrerer tegn
2	Forstår enkle tegn, mest konkrete tegn. (F.eks. forstår tegnet for bil, ball eller spise.)
3	Forstår tegn uten at konteksten hjelper til (F.eks. at barnet ikke kan se tingen som det snakkes om.) Forstår abstrakte tegn. (F.eks. tegnene”tisse”,”pause” mv.)
4	Forstår korte setninger på tegnspråk. (F.eks. beskjeder, ordre.)
5	Kan inngå i korte dialoger om ting som ikke er konkret nærværende og hverdagssetninger
6	Kan uten vansker delta i vanlig samtale på tegnspråk
7	Kan fullt ut delta i lengre og komplekse samtaler på tegnspråk om et kjent emne, forstå fortellinger på tegnspråk, aldersadekvate TV-programmer på tegnspråk og lignende uten problemer

Jesper Dammeyer, 2006.

Norsk oversettelse: Chris M. Aanondsen, 2013

**Table 10:** Norwegian version of the Sign Language Production Scale (SUS) VTP—Vurdering av tegnspråklig produksjon Sett et kryss ved nivået, som passer best på barnet

1	Barnet produserer ikke egentlige tegn. Bruker enkelte gester og pekninger
2	Barnet kan produsere enkelte vanlige tegn når konteksten hjelper til
3	Barnet kan tegne enkle handlingsforløp av minimum to-tre tegn. Tegnspråket er forståelig for personer som kjenner barnet godt
4	Barnet kan tegne setninger med flere enn tre tegn som ikke nødvendigvis er grammatisk korrekte. Enkel bruk av proformer. Tegnspråket er forståelig for personer som kan tegnspråk, men som ikke kjenner barnet
5	Barnet har et flytende og nesten konvensjonelt korrekt tegnspråk. Tegnspråket er lett forståelig for alle som kan tegnspråk. (Bruker f.eks. proformer og grammatisk ansiktsuttrykk kreativt.)

Jesper Dammeyer, 2006

Norsk oversettelse: Chris M. Aanondsen, 2013.

## Appendix 3

**Table 11:** Table B1 Missing values handled by multiple imputation

Variable	*n* (D)HH	Missing	%
Group	106	0	0.0
Gender	106	0	0.0
Age	106	0	0.0
Mother's education	106	20	18.9
Father's education	106	24	22.6
Nonverbal IQ	106	35	33.0
Age at detection	106	19	17.9
Aetiology of HL	106	20	18.9
Degree of HL	106	37	34.9
Additional impairment	106	20	18.9
SDQ-P	106	19	17.9
SDQ-NOR^1^	79	10	12.7
SDQ-NSL^2^	48	9	18.8
ILC-P	106	19	17.9
ILC-NOR	106	12	11.3
ILC-NSL^3^	59	13	22.0
CCC-2 GCC^4^	97	24	24.7
Spoken Language Skills	106	30	28.3
Sign Language Skills^3^	59	21	35.6

^1^20 DHH and HH children too young for SDQ self-report

^2^ total number of signing DHH children ages ≥ 9 years

^3^ total number of signing DHH

^4^ total (D)HH who speak in complete sentences
